# Effects of interpersonal psychotherapy on anxiety and alexithymia in mothers in the postpartum period: a randomized controlled trial

**DOI:** 10.1590/1806-9282.20241002

**Published:** 2024-12-16

**Authors:** Vesile Adiguzel, Ayse Okanli

**Affiliations:** 1 Yalova University, Faculty of Health Sciences, School of Nursing, Department of Psychiatric Nursing – Yalova, Turkey.; 2 İstanbul Medeniyet University, Faculty of Health Sciences, School of Nursing, Department of Psychiatric Nursing – İstanbul, Turkey.

**Keywords:** Postpartum, Anxiety, Alexithymia, Interpersonal psychotherapy, Psychiatric nursing

## Abstract

**OBJECTIVE:**

The aim of this study was to determine the effects of interpersonal psychotherapy on anxiety and alexithymia in mothers in the postpartum period.

**METHODS:**

This randomized controlled experimental study with a pre-test and post-test design was conducted to determine the effects of interpersonal psychotherapy on anxiety and alexithymia in mothers during the postpartum period. Data were collected from postpartum mothers who presented to the pediatric outpatient clinic in a private hospital in Istanbul at the pre- and post-tests between August 2022 and January 2023. The study was conducted between June 10, 2022, and June 23, 2023. In the study, data were obtained with the "Personal Information Form," "Beck Anxiety Inventory," and "Toronto Alexithymia Scale." This randomized controlled experimental study with the pre- and post-test designs was conducted in a private hospital in Istanbul, a province located on both sides of the Bosporus Strait in northwest Turkey, with 14 postpartum mothers. Of them, seven were assigned to the experimental group and seven to the control group. The participants in the experimental group underwent interpersonal psychotherapy. The participants in the control group underwent no intervention. In the analysis of the data, while repeated measures analysis of variance was used to compare the scale scores, Fisher's least significant difference test was used for multiple comparisons of the group*time interaction.

**RESULTS:**

The mean scores obtained from the "Beck Anxiety Inventory" and "Toronto Alexithymia Scale" by the participants in the experimental group before the interpersonal psychotherapy were 19.71±4.11 and 55.43±9.05, respectively, and 8.86±3.02 and 41.00±7.44, respectively, after the intervention, indicating that they decreased statistically significantly (p<0.05).

**CONCLUSION:**

The results of the study indicated that interpersonal psychotherapy reduced anxiety and alexithymia levels in mothers in the postpartum period. Based on this result, it is recommended that interpersonal psychotherapy be utilized in clinical practice.

## INTRODUCTION

Pregnancy and birth are unique experiences for mothers during which they experience bio-psychosocial changes; thus, they may be very fragile, especially psychologically^1–3^. During this period when fragility is intense, they may experience anxiety at levels ranging from normal to devastating levels^4^. Mother's being provided with the support she needs during pregnancy, birth, and postpartum by her spouse, family, environment, or health professionals is important for her to cope with anxiety. In several studies, it has been indicated that the mother goes through this process more comfortably if she is supported by her family in the postpartum process. Among psychosocial supports provided to the mother are support provided by her spouse or family members in baby care, help with housework, and emotional support^5^. Mothers who are not supported adequately in the postpartum period suffer from mood disorders and anxiety disorders at a high rate^3,6^. If the need for psychosocial support is met during this period, the negativities created by stress and anxiety in the mother reduce and her self-confidence improves, and if she is enabled to express her feelings and thoughts, her adaptation to her new roles and responsibilities is facilitated, and she enjoys improved satisfaction with life and quality life^7,8^. Receiving inadequate psychosocial support during the postpartum period can cause the mother to experience emotional problems such as depression, anxiety, and stress, and can even negatively affect the bond between the mother and baby, and her marriage^9^. Alexithymia, an emotional problem, is characterized by a decrease in the ability to identify, analyze, and express emotions, limited imagination, and externally oriented thinking. Alexithymia is defined as having difficulty understanding and interpreting the feelings of others, establishing and maintaining interpersonal relationships, and having a limited social environment and a decrease in social competence^10^. According to the results of several studies, there is a positive significant relationship between anxiety and alexithymia^11–13^. Therefore, it is important to support mothers, especially in the postpartum period, from a psychosocial perspective. The review of the literature revealed that interpersonal psychotherapy (IPT) is implemented to prevent depression in mothers in the postpartum period^14–16^.

Among psychosocial support received by mothers are individual competence, psychological coping, determination, ability to care for oneself, strengthening of communication, marital satisfaction, reduced risk of mental problems, and psychological well-being^5^. IPT is an interpersonal attachment theory-based and limited-term therapy focusing on the mother's interpersonal problems, aiming to improve her social support and interpersonal functioning, in case she experiences emotional problems and anxiety-related disorders^17^. IPT, which was initially conceptualized for "pure" unipolar depression, was also adapted to postpartum depression. It is stated that studies conducted on IPT performed in the perinatal period focus on pregnancy depression and postpartum depression^18^. The search for interventional studies in which anxiety and alexithymia resulting from the emotional problems that may arise in psychosocially fragile mothers in the postpartum process were addressed together revealed a gap in the literature.

## OBJECTIVE

The present study aimed to determine the effects of the IPT undergone by mothers in the postpartum period on their anxiety and alexithymia levels.

## METHODS

### Study model

This randomized controlled experimental study with a pre-test and post-test design was conducted to determine the effects of IPT on anxiety and alexithymia in mothers during the postpartum period.

### Place and time of the study

Data were collected from postpartum mothers who presented to the pediatric outpatient clinic in a private hospital in Istanbul at the pre- and post-tests between August 2022 and January 2023. The study was conducted between June 10, 2022, and June 23, 2023.

### The population and sample of the study

The population of the study consisted of 126 mothers with 1-month-old babies in the postpartum period who presented to the pediatric outpatient clinic of a private hospital in Istanbul. The G*power program was used to determine the sample size. In performing the power analysis, the study titled "The Effect of Group Social Work Application Based on Interpersonal Therapy on Psychosocial Functioning in Women Diagnosed with Major Depression" was utilized^19^. According to the results of the analysis, it was determined that the sample should include 14 mothers (7 in the experimental group and 7 in the control group) (α=0.05, β=0.95). However, due to the possibility of losses during the study, four more mothers were included as reserves (two in the experimental group and two in the control group). After the adequate sample size was determined, the randomization stage was started. The mothers included in the study were randomly assigned to the experimental and control groups. Since no losses occurred during the interviews, the reserved mothers were not included in the study.

### Criteria for inclusion in the research

- Being over 18 years of age,
- Being in the postpartum period,
- Scoring over 15 points on the Beck Anxiety Scale,
- Having a 1-month-old baby,
- Having an internet connection,
- Ability to use a computer or smartphone for online meetings.

### Exclusion criteria

- Being diagnosed with any psychiatric disease,
- Having received similar training/therapy approaches before,
- Not participating in more than 20% of the given application,
- Having received an additional psychiatric diagnosis during the approach.

### Randomization

**Figure 1:**
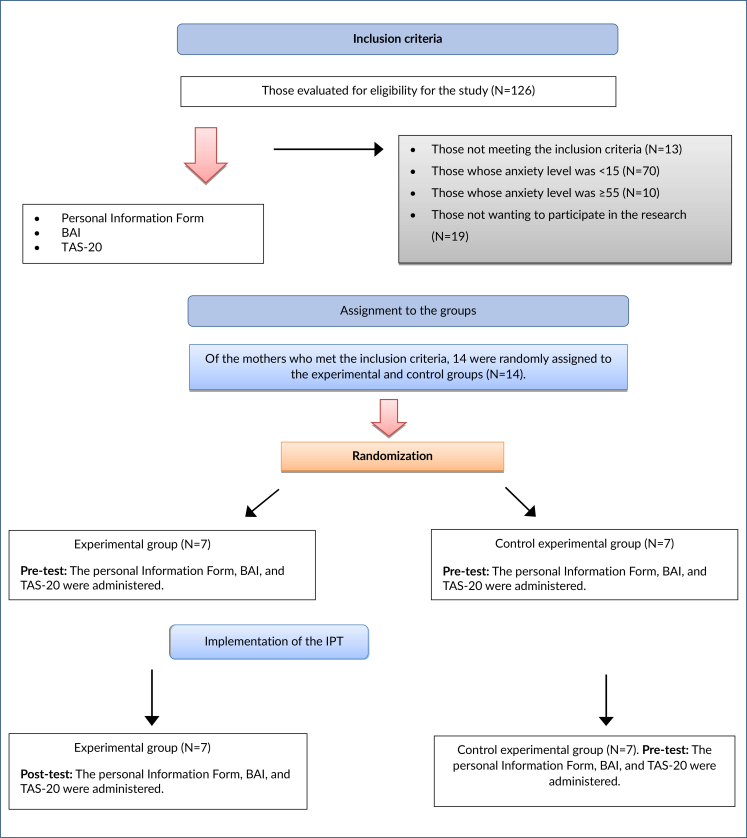
Consort diagram.

Randomization was performed using computer-based random sequence numbers (www.random.org). The participants were numbered in the order they were included in the study, and they were assigned to the experimental group or control group according to the random sequence numbers in the randomization list. All the individuals included in the sample were administered the Informed Consent Form, Personal Information Form, the Beck Anxiety Inventory, and the Toronto Alexithymia Scale before the study (Figure 1).

### Measurement tools used

Personal information form: The Personal Information Form developed by the researchers through a literature review was administered to question the participants’ characteristics such as age, the number of children, and the number of pregnancies.

### Beck Anxiety Inventory

The BAI developed by Beck is used to assess the level of anxiety and to find out the cognitive aspects of anxiety. The BAI consists of 21 items whose responses are rated on a 4-point Likert-type scale ranging from 0 to 3. Ulusoy et al. conducted the first validity and reliability study of the Turkish version of the BAI, and they calculated its test–retest reliability coefficient as 0.57. The score obtained from the overall BAI is used for assessment. The higher the score, the higher the level of anxiety is^20^. In the present study, the Cronbach's alpha coefficient of the BAI was calculated as 0.82.

### Toronto Alexithymia Scale-20

The scale developed by Bagby et al.^21^ initially consisted of 26 items, but then it was shortened to 20 items (Toronto Alexithymia Scale-20 [TAS-20]). Today the short form is frequently used. The validity and reliability study of the Turkish version of the TAS-20 was performed by Kose et al. The Cronbach's alpha values of the sub-dimensions were 0.82, 0.75, and 0.72, respectively, in Kose et al.'s study. The score for the overall TAS-20 is calculated by summing the scores of the items. The minimum and maximum possible scores that can be obtained from the scale are 20 and 100, respectively. High scores indicate that the level of alexithymia is high^22^. In the present study, the Cronbach's alpha coefficient of the overall TAS-20 was 0.89.

### Nursing intervention

#### Implementation of the interpersonal psychotherapy

The IPT program was prepared by the researcher who took training in interpersonal psychotherapy. The content of the program was presented to two psychiatrists and two psychiatric nurses to obtain expert opinion, and expert approval was obtained. The mothers participating in the study were informed about the IPT and were asked whether they wanted to participate in the study. Before the IPT was implemented, a program was planned for each mother in the experimental group. They participated in one 40–60-min session per week for 9–12 weeks. A different day and time were allocated to each mother to participate in the sessions. Because the mothers did not have anyone to look after their babies when they participated in the IPT program, not to cause them trouble in their home and family life, they were allowed to participate in the program together with their babies. To prevent the babies from interrupting the session, the program was held during the children's sleeping hours. The first session was carried out face-to-face, and the second and the following sessions were carried out one-on-one on the Internet through Zoom calls with smartphones. The mothers were informed about how the online sessions would be held after the first session. The researcher determined the mothers’ problem areas in the program. After the first interview, the researcher decided that the mothers’ "interpersonal conflict" and "role transitions" were the problem areas, and she told them that the focus would be on these problem areas^18^.

### Data analysis

Statistical analysis of the data was performed using the SPSS (Statistical Package for the Social Sciences) 22.0 program. The data of the study were presented as arithmetic mean and standard deviation for numerical variables and as frequency and percentages for categorical variables in the descriptive statistics. The chi-square test was used to compare the demographic characteristics of the participants according to the study groups. The Independent Samples t-test or Mann-Whitney U test was used to compare numerical variables in the study groups. The repeated measures analysis of variance method was used to compare the scale scores obtained at different times. Fisher's least significant difference (LSD) test was used for multiple comparisons of the group*time interaction. p<0.05 was accepted as the significance level.

### Ethical issues

Before the study was conducted, ethical approval was obtained from the Istinye University Human Research Ethics Committee (Protocol number: 22-92, Decision date: June 08, 2022). Institutional permission was obtained from the administration of the private hospital where the study was to be conducted. Written consent was obtained from the mothers who participated in the study. The study was conducted in accordance with the ethical standards established in the Declaration of Helsinki.

## RESULTS

The comparison of the postpartum mothers in the experimental and control groups in terms of the scores they obtained from the data collection tools at the pre-test was given in Table 1. There was no statistically significant difference between the experimental and control groups in terms of the scores they obtained from the scales (p>0.05).

**Table 1:** The comparison of the postpartum mothers in the experimental and control groups in terms of the scores they obtained from the Beck Anxiety Inventory and Toronto Alexithymia Scale at the pre-test.

Scales	Experimental group		Control group		t	p
	Mean±SD	Median (min–max)	Mean±SD	Median (min–max)		
Beck Anxiety Inventory Total	19.71±4.11	20 (13–24)	21.71±6.55	20 (16–35)	-0.684	0.507
Toronto Alexithymia Scale Total	55.43±9.05	56 (45–67)	59.71±17.92	55 (43–85)	-0.565	0.583

* p<0.05; independent samples t-test.

SD: standard deviation.

The comparison of the mean scores obtained from the Beck Anxiety Inventory (BAI) by the participants in the experimental and control groups is given in Table 2. The group*time interaction was statistically significant (p<0.05). While the mean scores obtained from the BAI by the participants in the experimental group before and after the intervention were 19.71±4.11 and 8.86±3.02, respectively, those obtained by the participants in the control group were 21.71±6.55 and 23.43±7.25, respectively. It was observed that while the mean BAI score did not differ statistically significantly over time in the control group (p>0.05), it statistically significantly decreased in the experimental group (p<0.05).

**Table 2:** Comparison of the mean scores obtained from the Beck Anxiety Inventory by the participants in the experimental and control groups.

Groups	N	BAI pre-test	BAI post-test	F	p-value
		$\bar{\text{X}}$ ±SD	$\bar{\text{X}}$ ±SD		
Experimental	7	19.71±4.11	8.86±3.02	8.801	0.012*
Control	7	21.71±6.55	23.43±0.25		
		F=23.676; p=0.001*		F_interaction_=44.763 p=0.001*	

* p<0.05; repeated measures analysis of variance.

BAI: Beck Anxiety Inventory; SD: standard deviation.

The comparison of the mean scores obtained from the Toronto Alexithymia Scale (TAS) by the participants in the experimental and control groups is given in Table 3. The group*time interaction was statistically significant (p<0.05). While the mean scores obtained from the TAS by the participants in the experimental group before and after the intervention were 55.43±9.05 and 41±7.44, respectively, those obtained by the participants in the control group were 59.71±17.92 and 61.14±18.19, respectively. It was observed that while the mean TAS score did not differ statistically significantly over time in the control group (p>0.05), it statistically significantly decreased in the experimental group (p<0.05).

**Table 3:** Comparison of the mean scores obtained from the Toronto Alexithymia Scale by the participants in the experimental and control groups.

Groups	N	TAS pre-test	TAS post-test	F	p-value
		$\bar{\text{X}}$ ±SD	$\bar{\text{X}}$ ±SD		
Experimental	7	55.43±9.05	41±7.44	2.666	0.128
Control	7	59.71±17.92	61.14±18.19		
		F=99.372; p=0.001*		F_interaction_=147.852 p=0.001*	

* p<0.05; repeated measures analysis of variance.

TAS: Toronto Alexithymia Scale; SD: standard deviation.

## DISCUSSION

The results of the present study in which the effects of interpersonal psychotherapy undergone by the participants in the experimental group on their anxiety and alexithymia levels were investigated were discussed in line with the literature. The fact that the mean score obtained from the BAI by the mothers who participated in the IPT decreased after the intervention and that there was no significant change in the mean scores obtained by the mothers in the control group who did not receive the intervention suggests that the intervention positively affected the mean BAI scores. It is estimated that this effect occurs because the IPT improved the mothers’ awareness of feelings of anxiety and because they started to express the feelings caused by anxiety appropriately and to display help-seeking behaviors. Given the attachment theory that forms the basis of IPT, it is thought that individuals building a good bond and establishing good communication with the people around them will be an important factor for them to feel safe and well.

The review of the literature demonstrated that the number of intervention studies conducted on interpersonal psychotherapy undergone by mothers in the postpartum period was limited^23^. Studies on interpersonal psychotherapy were mostly conducted with patients with postpartum depression^16^. In a study conducted with 185 women with recurrent depression who received interpersonal psychotherapy, there was a significant improvement in their depression symptoms^24^. The IPT was effective in the treatment of postpartum depression^25^.

The mean score obtained from the TAS by the mothers who participated in the IPT decreased after the intervention, and there was no significant change in the mean scores obtained by the mothers in the control group who did not receive the intervention, which suggests that the intervention positively affected the mean TAS scores. This was probably because the IPT helped mothers focus on themselves by increasing their problem-solving skills and developing their ability to express and define their emotions.

The search for studies in which the effects of interpersonal psychotherapy on the alexithymia levels of mothers in the postpartum period were investigated revealed a gap in the literature. Most of the studies were descriptive in nature and were conducted to determine alexithymia^12,26^. According to the results of the present study, the IPT was effective on alexithymia.

Thus, it can be stated that the IPT applied in the present study can contribute to the treatment of alexithymia. According to the results of the present study, the mean scores obtained from the overall BAI and TAS by the postpartum mothers in the experimental group decreased after they underwent the IPT. Thus, it can be said that the IPT may be effective in the treatment of anxiety and alexithymia in mothers in the postpartum period. Additionally, IPT is recommended to be used in clinical practice.

### Limitations and generalizability of the study

The results obtained from the present study are applicable only to the mothers who presented to the pediatric outpatient clinic of a private hospital in a province; thus, they cannot be generalized to all mothers. The findings of the present study are limited only to the data obtained with the measurement tools used. The results of the study are limited only to the analyses obtained by the statistical methods used. The first month observations of the study could not be conducted due to the earthquake that occurred in Turkey on February 6, 2023.
